# Hormone Receptor-Status Prediction in Breast Cancer Using Gene Expression Profiles and Their Macroscopic Landscape

**DOI:** 10.3390/cancers12051165

**Published:** 2020-05-05

**Authors:** Seokhyun Yoon, Hye Sung Won, Keunsoo Kang, Kexin Qiu, Woong June Park, Yoon Ho Ko

**Affiliations:** 1 Department of Electronics Eng., College of Engineering, Dankook University, Yongin-si 16890, Korea; syoon@dku.edu (S.Y.); qiukexin95@naver.com (K.Q.); 2 Department of Internal Medicine, College of Medicine, The Catholic University of Korea, Seoul 06591, Korea; woncomet@catholic.ac.kr; 3 Department of Microbiology, College of Natural Sciences, Dankook University, Cheonan-si 31116, Korea; kangk1204@dankook.ac.kr; 4 Department of Molecular Biology, College of Natural Sciences, Dankook University, Cheonan-si 31116, Korea; parkwj@dku.edu; 5 Cancer Research Institute, College of Medicine, The Catholic University of Korea, Seoul 06591, Korea

**Keywords:** breast cancer, intrinsic subtype, hormone receptor-status prediction, gene expression profile, LASSO regression

## Abstract

The cost of next-generation sequencing technologies is rapidly declining, making RNA-seq-based gene expression profiling (GEP) an affordable technique for predicting receptor expression status and intrinsic subtypes in breast cancer patients. Based on the expression levels of co-expressed genes, GEP-based receptor-status prediction can classify clinical subtypes more accurately than can immunohistochemistry (IHC). Using data from The Cancer Genome Atlas Breast Invasive Carcinoma (TCGA BRCA) and Molecular Taxonomy of Breast Cancer International Consortium (METABRIC) datasets, we identified common predictor genes found in both datasets and performed receptor-status prediction based on these genes. By assessing the survival outcomes of patients classified using GEP- or IHC-based receptor status, we compared the prognostic value of the two methods. We found that GEP-based HR prediction provided higher concordance with the intrinsic subtypes and a stronger association with treatment outcomes than did IHC-based hormone receptor (HR) status. GEP-based prediction improved the identification of patients who could benefit from hormone therapy, even in patients with non-luminal breast cancer. We also confirmed that non-matching subgroup classification affected the survival of breast cancer patients and that this could be largely overcome by GEP-based receptor-status prediction. In conclusion, GEP-based prediction provides more reliable classification of HR status, improving therapeutic decision making for breast cancer patients.

## 1. Introduction

Breast cancer is a highly heterogeneous disease that involves several complex molecular networks [1,2,3,4,5,6,7]. Breast cancer can be classified into different subtypes that have distinct clinical behaviors and prognoses and that require different treatment strategies. Therefore, accurate classification of breast cancer subtypes is crucial for personalized disease management and for improving patient outcomes [8,9]. The clinical subtypes of breast cancer are traditionally defined based on the expression status of three receptors: estrogen receptor (ER), progesterone receptor (PR), and human epidermal growth factor receptor 2 (HER2) [10,11]. Thus, the clinical subtypes are classified according to protein expression, as determined by immunohistochemistry (IHC), as hormone receptor positive (ER and/or PR positive), HER2 positive, or triple-negative breast cancer (ER, PR, and HER2 negative). Breast cancer is a hormone-driven malignancy that expresses various sex steroid receptors such as ER beta, PR, and androgen receptor, in addition to the classical hormonal marker ER alpha [12,13]. These sex steroid receptors positively or negatively modulate ER signaling and stimulate or inhibit cellular proliferation via interactions with other signaling pathways [12,13]. The recent discovery of the luminal androgen receptor subtype of triple-negative breast cancer has highlighted the importance of these sex steroid receptors in breast cancer classification and therapeutic decision making [13]. 

With the advent of high-throughput technologies for gene expression analysis, new molecular subtypes of breast cancer have been described, considering that systematic investigation of the expression patterns of thousands of genes and their phenotypic correlations could improve breast cancer classification. In the early 2000s, Perou et al. identified a distinctive molecular portrait of breast cancer based on variations in tumor gene expression patterns and hierarchical clustering [14,15], they described a molecular classification system for breast carcinoma consisting of five intrinsic subtypes: luminal A, luminal B, HER2-enriched, basal-like, and normal breast-like tumors. Several subsequent studies showed similar intrinsic molecular classifications, despite slight differences in the naming and numbers of categories and genes [9]. Four major classes distinct from normal breast-like tumors are currently widely accepted. The clinical significance of these intrinsic breast cancer subtypes has been highlighted by their ability to predict treatment response and prognosis [4,5,6,7,16,17,18,19,20,21,22,23]; hence their use in clinical practice has increased over recent years. Currently, several gene-signature tests based on microarray or quantitative real-time PCR (qRT-PCR) are commercially available [9,24,25].

The clinicopathological surrogate definitions of the intrinsic breast cancer subtypes were endorsed by the 2013 St. Gallen Consensus Recommendations [26]. Luminal A breast cancer is ER and PR positive and HER2 negative and expresses low levels of the protein Ki-67. Luminal B breast cancer is ER positive and either HER2 positive or HER2 negative, with high levels of Ki-67. The HER2-enriched subtype is ER and PR negative and HER2 positive, and the basal-like subtype is ER and PR negative and HER2 negative (triple-negative breast cancer) [27,28,29]. As clinical features of the four intrinsic breast cancer subtypes have been extensively studied in the last few years, discordance has been reported between IHC-based clinical subtypes and intrinsic subtypes in approximately 20%–50% of cases [20,30,31]. This discordance might be due to intratumoral heterogeneity and/or measurement inaccuracies in subtype profilers, i.e., IHC analysis for ER/PR status and fluorescence in situ hybridization (FISH) analysis for HER2 status. These inconsistencies could result in administration of the wrong treatment, subsequently leading to poor survival [32]. Therefore, accurate identification of receptor status or the intrinsic breast cancer subtype is of high clinical importance. 

Recently, multi-omics technologies [33], miRNA profiling [34] and principle component analysis-based iterative PAM50 subtyping [35] have helped to improve the accuracy of breast cancer subtype classification. However, inconsistencies due to measurement noise remain a challenge in this classification, especially for tumors with receptor expression levels at the boundary between positive and negative [35]. With the development of next-generation sequencing (NGS) technologies, the cost of gene expression profiling (GEP) based on RNA-seq is rapidly decreasing, making it possible to characterize several clinical and molecular features concurrently using RNA-seq-based GEP at a very low cost [36,37]. Prediction of the intrinsic subtype and receptor status (ER, PR, or HER2) in breast cancer using RNA-seq-based GEP would increase the clinical usefulness of RNA-seq technologies in breast cancer. In this study, we investigated whether RNA-Seq-based GEP could be used for a better prediction of the status of the three receptors by assessing the survival outcomes under the GEP-based prediction, thereby improving therapeutic decision making.

## 2. Results

### 2.1. Identification of Predictor Genes

In this study, IHC-based characterization of receptor status in breast cancer was refined by using co-expressed predictor genes. First, predictor genes were identified; seven genes were selected for ER status prediction, six for PR, and four for HER2 (Table 1). As expected, the *ESR1, PGR,* and *ERBB2* genes, which encode the ER, PR, and HER2 proteins, respectively, were among the predictor genes. Model training and receptor-status prediction were then performed using the selected genes. The mismatch rate reported in Table 1 is the percentage of cases in which the IHC-based status differed from the predicted status. Among the predictor genes, *TFF1* and *NAT1* were included in an eighteen-gene set previously reported to predict sensitivity to hormone therapy [38,39].

### 2.2. Macroscopic Landscape

**Figure 1:**
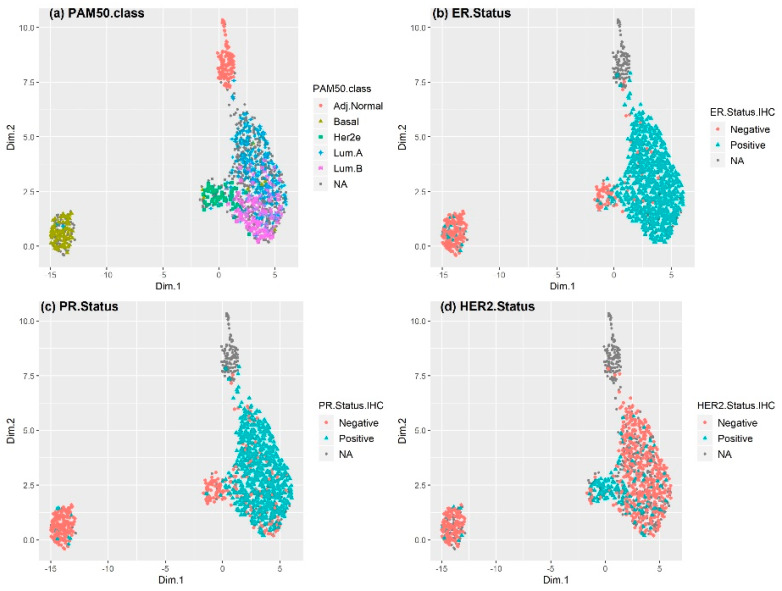
Uniform manifold approximation and projection (UMAP) plot showing the receptor status in the TCGA Breast Invasive Carcinoma (BRCA) cohort. Each point represents a sample; the color of the spots corresponds to the (**a**) subtype (PAM50 class), (**b**) ER status, (**c**) PR status, and (**d**) HER2 status of the sample. The tumor subtype, as well as the status of ER, PR, and HER2, were based on the available clinical data. Gray points are samples with no available clinical information. A small percentage of the luminal A and B subtypes were ER-/PR- and HER2+. Such discordances were also observed in some breast cancer patients with the HER2-enriched and basal-like subtypes. Although most HER2+ and HER2-enriched subtype samples overlapped, some HER2-enriched subtype samples were found to be HER2-breast cancer and to exhibit basal-like subtype features. Some samples were ER+ and/or PR+, representing a luminal subtype.

Figure 1 shows uniform manifold approximation and projection (UMAP) plots [40] for receptor status in The Cancer Genome Atlas Breast Invasive Carcinoma (TCGA BRCA) cohort. Each point represents a sample; the color of the spots corresponds to the (a) subtype (PAM50 class), (b) ER status, (c) PR status, and (d) HER2 status of the sample. Receptor status (ER, PR, or HER2) was provided in the original clinical data based on IHC. The expression of 100 genes selected by LASSO was used to obtain the two-dimensional UMAP projection. The luminal A and B subtypes were mostly HER2- and either ER+ or PR+. However, a small percentage of the luminal A and B subtypes exhibited ER-, PR-, and HER2+. Some patients with HER2-enriched or basal-like subtype breast cancer also showed some level of discordance, as some HER2-enriched and basal-like subtype samples were ER+ or PR+. Although most HER2+ and HER2-enriched subtype samples overlapped, some HER2-enriched subtype samples were found to be HER2-breast cancer that exhibited basal-like subtype features. As only eight patients exhibited normal breast-like breast cancer in the TCGA dataset, they were not considered in our analyses.

On the other hand, the HER2-enriched subtype samples were ER+ and/or PR+, representing a luminal subtype. The UMAP plot of the METABRIC dataset revealed a similar macroscopic landscape (Figure S1). Considering that the distance between samples (points) in the UMAP projection is only an approximation of the relative distance in their gene expression profiles and that the receptor status was not clearly defined for all samples, Figure 1 implies that IHC/FISH-based characterization of receptor status might result in inaccuracies in breast cancer subtype classification.

**Figure 2:**
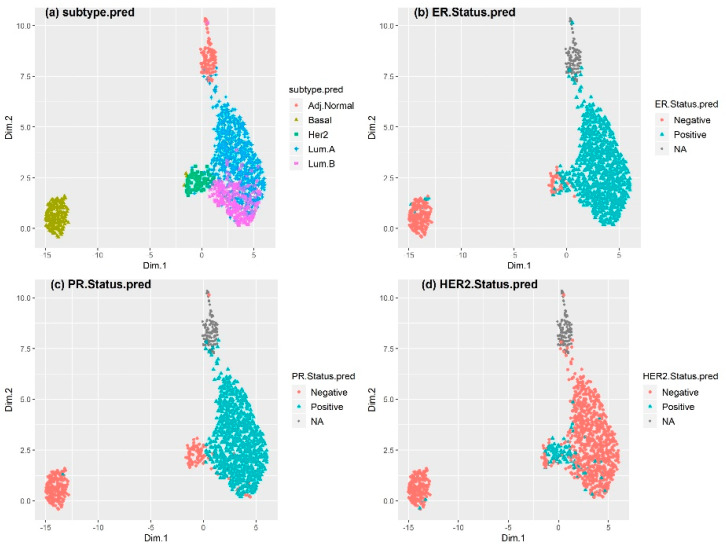
UMAP plot showing gene expression profiling (GEP)-based receptor status in the TCGA BRCA cohort. Each point represents a sample; the color of the spots corresponds to the (**a**) subtype, (**b**) ER status, (**c**) PR status, and (**d**) HER2 status of the sample. GEP-based prediction was used to determine the subtype, as well as the status of ER, PR, and HER2. Compared to the case with IHC-based approaches, the predicted status of ER, PR, and HER2 was mostly in accordance with the corresponding pattern of receptor status for basal-like, luminal A, and luminal B. In contrast, HER2-enriched subtype tumors were highly heterogeneous.

Figure 2 shows the same UMAP plot based on the predicted values obtained by the linear classifiers. Compared with IHC-based receptor-status characterization, the predicted status was more consistent with the intrinsic breast cancer subtype classification, especially for the basal-like and luminal subtypes. Most of the luminal subtypes were ER+ and PR+, and the numbers of ER+ or PR+ samples in the basal-like subtype were much smaller than after IHC-based status characterization. The UMAP plot for the METABRIC dataset based on the predicted receptor status (Figure S2) led to the same conclusions, except for PR status, which was not IHC-based in the METABRIC dataset.

### 2.3. GEP-Based Receptor-Status Prediction is Reliable for the Luminal and Basal-Like Subtypes

To quantify discordance between the intrinsic subtype and the clinical subtype defined by HR and HER2 status, for each intrinsic subtype, we compared the numbers of positive and negative instances of HR and HER2 status based on IHC with the numbers obtained using GEP-based prediction in the TCGA and METABRIC datasets (Table 2). The rates of discordance for the basal-like, luminal A, and luminal B subtypes were lower using GEP-based prediction than using IHC-based status characterization. Specifically, most samples of the luminal A and B subtypes were characterized as HR+ by GEP-based prediction (except for two samples in the TCGA BRCA cohort), while some luminal A and luminal B breast cancer samples were characterized as HR- based on IHC. In breast cancer patients with the basal-like subtype, a smaller percentage of tumors was determined to be HR+ using GEP-based prediction (10% in TCGA and 13% in METABRIC) than when using IHC-based characterization (17% in TCGA and 20% in METABRIC) (Tables S1 and S2).

On the other hand, considerable discordance was observed in the receptor status of HER2-enriched subtype breast cancer patients using both IHC-based characterization and GEP-based prediction. Only 37% and 23% of patients with HER2-enriched subtype breast cancer were HR-/HER2+ in the METABRIC and TCGA datasets, respectively. Furthermore, 17% and 18% of tumors were triple negative, and 25% and 9% were luminal-like (HR+ and HER2−) in the METABRIC and TCGA datasets, respectively. Similar findings were obtained for IHC-based characterization of HR and HER2 status. 

In summary, GEP-based prediction was more concordant with the typical receptor-status pattern of the intrinsic subtypes of patients with the basal-like, luminal A, and luminal B subtypes. However, this does not necessarily mean that receptor-status prediction based on GEP is more accurate than IHC-based characterization. The only way to verify the accuracy of the status predictions is to assess the differences in clinical outcomes among the different clinical subtypes defined by the status of the three receptors.

### 2.4. GEP-Based Receptor-Status Prediction Had Higher Prognostic Significance in Terms of Patient Survival

To verify the accuracy of the receptor-status predictions, survival outcomes for various combinations of HR and HER2 status were compared. The significance of the prognostic value of the predicted and IHC-characterized HR and HER2 status was compared. Separate survival analyses were performed in the following four patient groups:

(a) HR+ (either ER+ or PR+) group: this group benefited from hormone therapy. According to the stage and clinical characteristics, these patients often received a combination of hormone therapy and chemotherapy. For survival analysis, the patients in this group were stratified based on administration of hormone therapy.
(b) Hormone therapy group: to confirm the benefit of hormone therapy for HR+ patients, only those who received hormone therapy, with or without chemotherapy, were selected, and the survival of HR+ patients was compared to that of HR– patients.
(c) HR+/non-luminal subtype group: as shown in Table 2, there were small percentages of HR+ patients among patients with the HER2-enriched and basal-like subtypes. Hence, we assessed whether breast cancer patients with the HR+ non-luminal subtype benefited from hormone therapy.
(d) HER2+ group: breast cancer patients with the HER2+ subtype benefited from anti-HER2 targeted molecular therapy (TMT). We assessed the survival of HER2+ breast cancer patients based on TMT. As no information regarding TMT was available in the METABRIC dataset, this analysis was performed only for the TCGA BRCA cohort.

**Figure 3:**
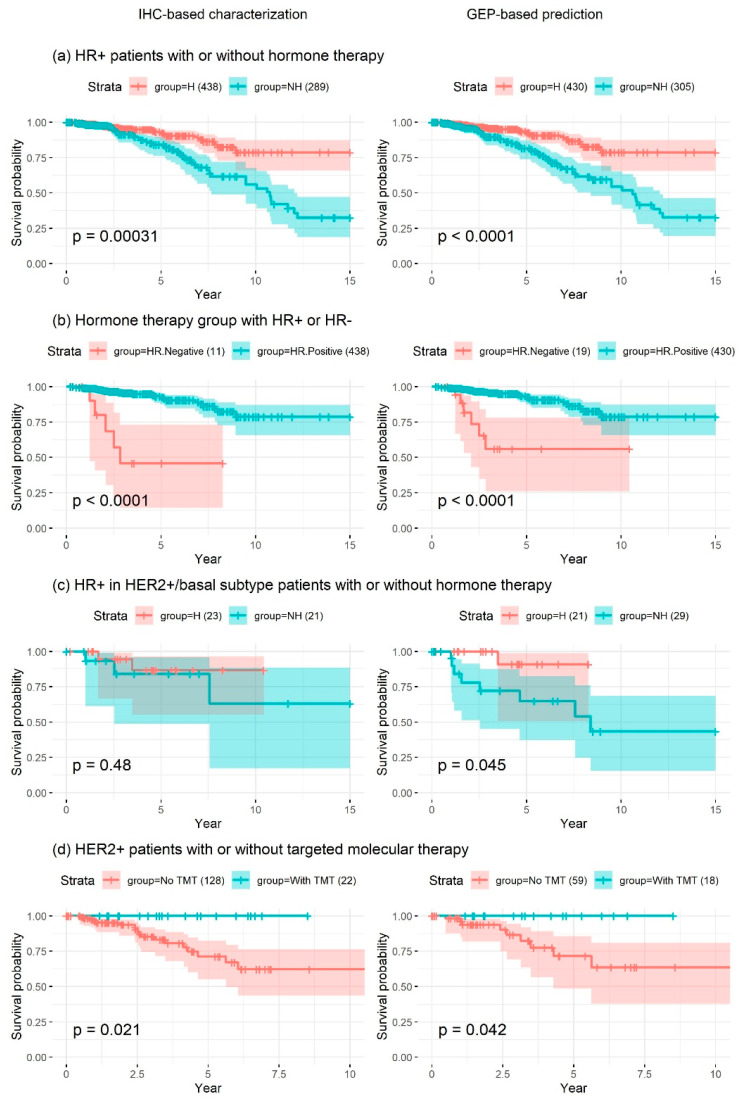
Kaplan–Meier survival analysis of patients from the TCGA dataset using IHC-based (left panel) or GEP-based (right panel) receptor status in the following four patient groups: (**a**) HR+ (either ER+ or PR+) group, (**b**) hormone therapy group, (**c**) HR+/non-luminal subtype group, and (**d**) HER2+ group. Patients were stratified to those who received hormone therapy (H) and those who did not (NH), or those who received targeted molecular therapy (With TMT) and those who did not (No TMT). (**a**) GEP-based receptor status prediction had higher prognostic significance in terms of patient survival compared to IHC-based HR status. (**b**) IHC-based receptor-status characterization was found to be more accurate than GEP-based receptor-status prediction. However, the numbers of samples in the test group (HR– patients) for receptor-status characterization based on IHC and GEP were only 11 and 19, respectively. (**c**) IHC-based receptor status had no significant prognostic value, in contrast to GEP-based receptor-status prediction. (**d**) The statistical significance of IHC-based receptor-status characterization indicated higher prognostic value. However, the numbers of patients with IHC-based receptor-status data in the test group (HER2+ patients with targeted molecular therapy, TMT) were only 22 under IHC and 18 under GEP, and all patients who received TMT survived; hence, the hazard ratio could not be precisely determined. H, hormone therapy; NH, no hormone therapy; TMT, targeted molecular therapy.

**Figure 4:**
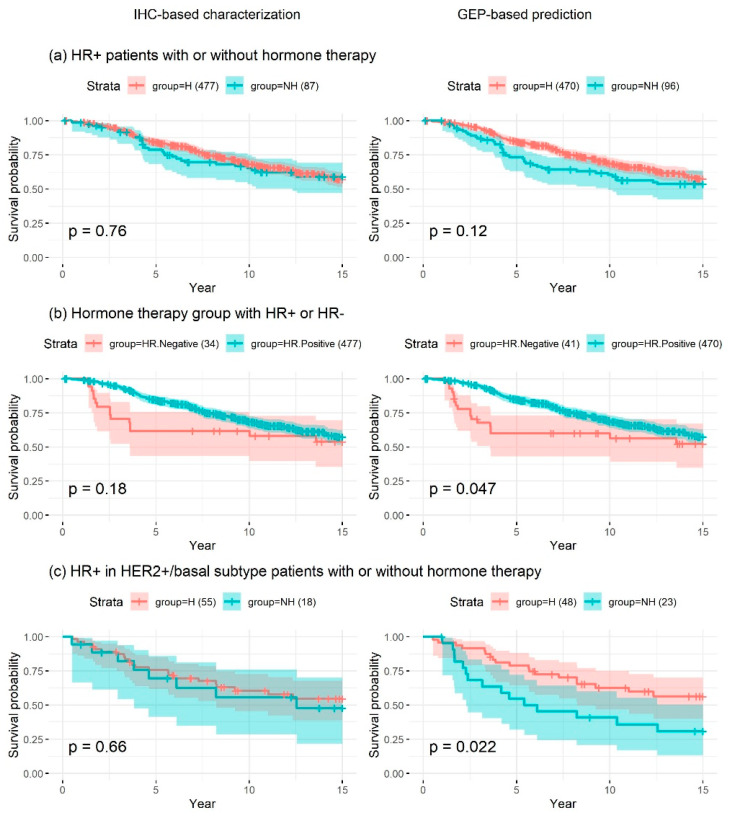
Kaplan–Meier survival analysis in patients of the METABRIC dataset with a pathological stage of II or III (excluding pathological stage I). The analysis was performed using IHC-based receptor (left panel) or GEP-based receptor (right panel) status in the following four patient groups: (**a**) HR+ (either ER+ or PR+) group, (**b**) hormone therapy group, and (**c**) HR+/non-luminal subtype group. Patients were stratified to those who received hormone therapy (H) and those who did not (NH). GEP-based receptor-status prediction had higher prognostic significance in terms of patient survival compared to traditional IHC-based receptor-status characterization. H, hormone therapy; NH, no hormone therapy.

Among patients in the TCGA BRCA cohort, GEP-based receptor-status prediction provided a higher hazard ratio with higher significance in HR– patients (a), implying that GEP-based receptor-status prediction had higher prognostic value than traditional IHC-based HR status characterization. On the other hand, in the hormone-therapy group (b), IHC-based receptor-status characterization was found to be more accurate than GEP-based receptor-status prediction. However, the numbers of samples in the test group (HR-patients) were only 11 and 19 for receptor-status characterization based on IHC and GEP, respectively. Among patients with HR+ non-luminal subtype breast cancer (c), IHC-based receptor status had no significant prognostic value, in contrast to GEP-based receptor-status prediction. This finding highlighted that HR+ breast cancer patients benefited from hormone therapy, even if they were diagnosed with non-luminal subtype tumors. Among HER2+ patients (d), IHC-based receptor-status characterization exhibited higher prognostic value when considering only the *p*-value. However, the numbers of patients with IHC-based receptor-status data in the test group (HER2+ patients with TMT) were only 22 and 18 based on IHC and GEP, respectively, and all patients that received TMT survived; hence, the hazard ratio could not be precisely determined (Figure 3 and Table 3). The small differences between *p* values shown in Figure 3b,d may have been caused by the very small target sample sizes, whereas that shown in Figure 3a is likely meaningful, since the sample sizes were large (control group, ~430; test group, 300). The difference between *p* values shown in Figure 3c was sufficiently large to conclude that the GEP-based status was more accurate than the IHC-based status, regardless of the small sample size. Survival analyses in the METABRIC cohort (excluding patients with a pathological stage of I) showed similar findings, implying that GEP-based receptor-status prediction had higher prognostic significance in terms of patient survival compared to traditional IHC-based receptor-status characterization (Figure 4 and Table 3).

### 2.5. Patients with Non-Matching Receptor Status Had Significantly Worse Survival

**Figure 5:**
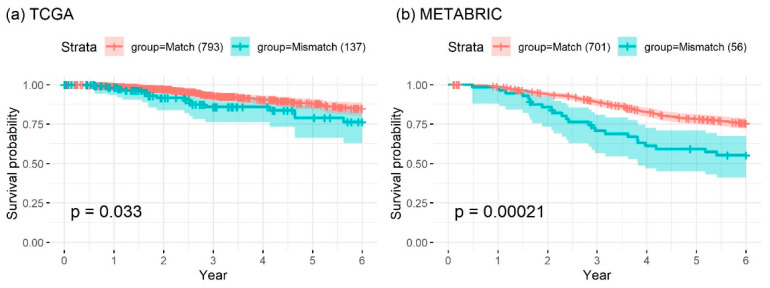
Kaplan–Meier survival analysis of patients in the (**a**) TCGA BRCA cohort and (**b**) METABRIC dataset with matching and non-matching receptor status. The hazard ratios of patients with non-matching status were 0.6 for the TCGA BRCA cohort and 0.79 for the METABRIC dataset.

The type of adjuvant therapy is based mainly on the status of the three receptors. Hence, accurate characterization of receptor status is of high clinical importance. As shown in Figure 5, patients with matching receptor status had longer overall survival (OS) compared to those with non-matching status (hazard ratios 0.6 and 0.79 for the TCGA BRCA and METABRIC cohorts, respectively). Assuming higher accuracy for GEP-based receptor-status prediction, these results highlight the impact of inappropriate treatment due to errors in receptor-status characterization. Although it is unlikely that GEP-based receptor-status prediction is 100% accurate, it can identify patients who can benefit from hormone therapy more reliably than the traditional IHC-based method.

## 3. Discussion

IHC-based assessment of the expression of a specific protein is undoubtedly an important tool for detecting biomarkers in clinical practice. However, this procedure entails severe limitations, including variations in the IHC procedure that can influence the results and their interpretation. As an alternative, biomarker characterization could be performed at the mRNA level; unfortunately, high mRNA levels do not necessarily translate into high levels of the corresponding protein. Additionally, characterization based solely on the expression levels of a single gene or protein inevitably entails the risk of noise. To overcome these limitations, we considered the potential use of GEP-based receptor-status prediction for molecular characterization of breast cancer subtypes. Changes in the expression of a gene should be reflected in those of co-expressed genes; therefore, prediction based on the expression of correlated genes may outperform molecular characterization based on a single gene.

In the era of biomarker-assisted targeted therapy, the method used to assess biomarker expression is crucial, as it can improve the prognosis for patients with breast cancer and other malignancies. Several challenges remain to be overcome in biomarker-assisted targeted therapies, such as IHC-determined borderline HR-positivity, equivocal HER2 amplification, and discordance between IHC-based subtypes and intrinsic subtypes. Previous studies have shown significant discordance between clinical subtypes and intrinsic subtypes, which affects the prognosis of breast cancer patients. Kim et al. reported that discrepancies between the IHC-based subtype and the intrinsic subtype were associated with poor survival, highlighting the limitations of current IHC-based classification methods [32]. A previous study reported that 27% of ER+ tumors were non-luminal breast cancer, and that the intrinsic subtype added significant prognostic and predictive values to standard clinical markers [20]. A subsequent study found that 58.3% of the ER+/HER2 subcohort had the luminal A subtype and 77.3% of the ER-/HER2 subcohort had the basal subtype [30]. Among the HER2+ breast cancer cases, 51% showed the HER2-enriched subtype, and the complete pathologic response rate to HER2-targeted neoadjuvant therapy was significantly higher in the HER2-enriched subtype than in the luminal A and B subtypes [31]. Consistent with these results, we confirmed the poor survival of patients with non-matching subgroup classifications in both the TCGA and METABRIC datasets. These results emphasize the clinical importance of establishing more accurate classification methods. Herein, we evaluated the concordance between the intrinsic subtype and the predicted status of ER, PR, and HER2 using GEP. We found a higher concordance rate between the intrinsic subtype and GEP-based receptor-status prediction compared to receptor status as characterized by IHC. This was consistent in all breast cancer subtypes except for the HER2-enriched subtype. These findings imply that GEP-based HR status prediction could be a promising alternative approach to IHC.

Both IHC-based receptor-status characterization and GEP-based status prediction resulted in considerable discordance between HER2-positivity and the HER2-enriched subtype. Although the HER2-enriched subtype is the predominant type of HER2+ breast cancer, three other subtypes exist. A recent study analyzing data from four prospective neoadjuvant trials reported that the percentages of the luminal A, luminal B, HER2-enriched, and basal-like subtypes among HER2+ breast cancer patients were 24%, 20%, 47%, and 9%, respectively [41]. This finding may be partly explained by high intratumoral heterogeneity. Previous genomic analyses have revealed that HER2+ breast cancer is extremely clinically and biologically heterogeneous [42,43]. The HER2-enriched subtype is also highly heterogeneous, rendering IHC/FISH- and PAM50-based subtyping challenging. 

Furthermore, the HER2-enriched subtype can have a distinctive transcriptional landscape independent of HER2 amplification. Analyses in TCGA showed that the HER2-enriched subtype was characterized by the highest number of DNA mutations, including in TP53 and PIK3CA [28]. Recently, Daemen A et al. performed genomic and transcriptomic profiling of HER2-enriched tumors; they concluded that HER2 was not a cancer subtype but rather a pan-cancer phenomenon and that HER2+ tumors are hormonally driven [44]. Even though further stratification of HER2-enriched breast cancer might be beneficial, it might be difficult to achieve further characterization based on GEP. To overcome the limitations of macroscopic GEP, different microscopic prediction approaches could be used, including laser dissection of specimens for transcriptomic analyses of subcellular populations, precise reconstruction of transcriptome data and use of single-cell RNA-seq. These approaches might achieve more in-depth characterization of the molecular subtypes.

To investigate the clinical relevance of GEP-based prediction of ER, PR, and HER2 receptor status, we performed survival analysis of HR+ patients who did or did not receive hormone therapy, as well as of HR+ and HR– patients treated with hormone therapy. GEP-based receptor-status prediction showed a more significant association between treatment outcomes and HR status compared to IHC-based receptor-status characterization. Of note, some benefit was achieved from hormone therapy by patients who were identified as HR+ non-luminal breast cancer using GEP-based prediction, in contrast to when IHC-based HR status characterization was performed. These results imply that GEP-based receptor-status prediction can better identify patients who can benefit from hormone therapy, even in patients with non-luminal subtype breast cancer. Some studies have shown that adjuvant or palliative hormone therapy is less effective in patients with HR+ breast cancer of the non-luminal subtype [45,46]. However, there is limited evidence regarding which HR+ non-luminal breast cancer patients will benefit from hormone therapy. Future studies are needed to determine whether GEP-based receptor-status prediction can address these clinically important questions. In contrast to the HR status, we did not observe improvement in HER2 status prediction; this may be attributed partially to the small number of patients who received targeted molecular therapy for HER2.

## 4. Materials and Methods

**Figure 6:**
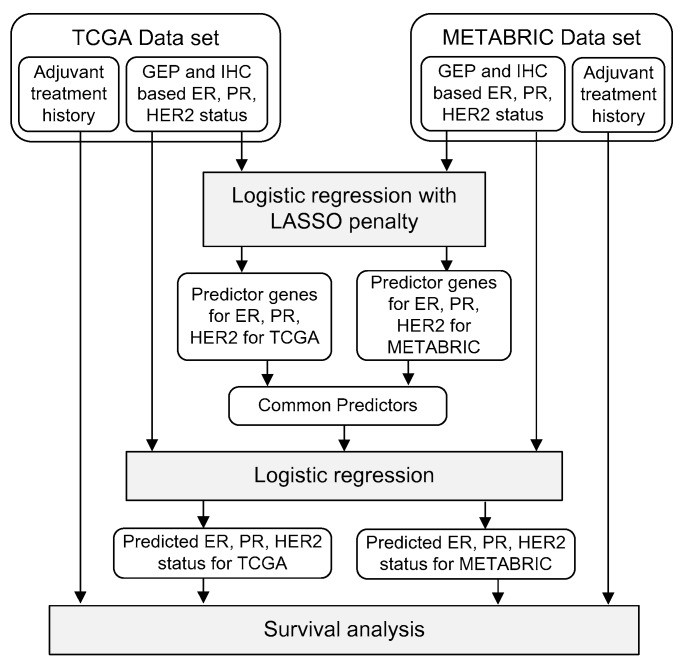
Workflow of gene selection, model training, receptor-status prediction, and survival analysis.

The theoretical basis for using GEP to improve predictions of hormone receptor status (i.e., ESR1, PGR, and ERBB2 gene expression statuses) is that other genes have highly correlated expression levels that indirectly reflect the expression levels of the target genes. Although the measured expression levels of these genes contain independent noise, this noise may be reduced according to the correlation between relevant genes and their expression levels. Several machine learning techniques can be applied for this purpose, e.g., extreme gradient boost (XGB) [47] or support vector machine (SVM) [48] methods. In this study, we used logistic regression with a LASSO penalty; this approach is suitable for the prediction of hormone receptor status as well as the selection of co-expressed predictor genes. The workflow of this study is shown in Figure 6. Our analyses were performed in three steps. First, we identified common predictor genes from two different gene-expression datasets. Second, we predicted ER, PR, and HER2 status based on the shared predictor genes. Finally, we compared survival outcomes according to IHC-based and GEP-based predictions of receptor status.

### 4.1. Datasets

For this study, we used breast cancer patients’ gene-expression-profile and clinical data acquired from The Cancer Genome Atlas (TCGA) [http://firebrowse.org/] and the Molecular Taxonomy of Breast Cancer International Consortium (METABRIC) databases [https://www.cbioportal.org/] [27]. Both datasets include information on the history of adjuvant treatment, which was a critical element in the survival analyses performed in this study. A summary of the data contained in the two datasets is shown in Table 4.

The TCGA BRCA dataset contained data from tumor samples (*n* = 1092 patients) and adjacent normal tissues (*n* = 112 patients). The METABRIC dataset contained data from 2506 tumor samples, including GEP data from 1904 patients. The TCGA and METABRIC datasets also contained clinical data, including ER, PR, and HER2 status, as well as histories of surgery, radiation-therapy, and drug treatments; however, clinical data were not available for all of the patients. Information regarding the tumor subtype was available for some samples in the TCGA BRCA dataset; PAM50 mRNA profile information was available for 523 of 1092 patients [26]. To ensure consistency between the two datasets, information on ER and HER2 status as determined by IHC was used for patients in the METABRIC dataset. Non-IHC-based PR status was used for the METABRIC cohort because the PR status was not assessed by IHC in these patients.

### 4.2. Prediction Model and Gene Selection

Based on GEP and the status of the three receptors, logistic regression with LASSO penalty was performed in a supervised mode to identify predictor genes for each of the two datasets. This analysis was performed using the R package glmnet [49,50,51]. In the TCGA BRCA dataset, the expression levels of 17,202 genes were log2-transformed and normalized. In the METABRIC dataset, already normalized mRNA expression data were used. To identify the common predictor genes and minimize overfitting-related errors, LASSO penalty weights were selected for a set of predefined genes (e.g., 10, 20, 40, and 60), and for each number, the penalty weight that led to the closest number of selected genes was chosen. This approach was conducted separately for the TCGA and METABRIC datasets. Common predictor genes between TCGA and METABRIC were then identified to avoid dataset-related dependencies. After inspecting the overall number of shared genes, 40 genes were selected; these contained 7, 6, and 4 common predictor genes for ER, PR, and HER2, respectively, as summarized in Table 1. Subsequently, logistic regression was performed again to train the models for ER, PR, and HER2 status prediction for both TCGA and METABRIC. The mismatch rate was obtained by fivefold cross-validation.

Pairwise correlations of gene-expression levels between the selected genes are shown in Figures S3, S4, and S5. Of note, PR predictor genes included *ESR1* and *AGR3,* which were also ER predictor genes. Furthermore, among the four HER2 predictor genes, *CPB1, GSTT1,* and *PROM1* showed only small correlations with ERBB2, implying that HER2 status prediction was determined predominantly by *ERBB2.*

### 4.3. Survival Analysis for Accuracy Evaluation and Sample Selection 

The survival analyses were performed for various group/condition pairs; significance (*p*-value) was used as an accuracy criterion. Cox’s proportional hazard model was used to determine overall survival [52]; the analysis was repeated using the IHC-based status and the predicted status. For the survival analysis based on IHC-based receptor status, we used those samples for which IHC-based receptor status was available. For the survival analysis based on predicted-receptor status, we used the same set of samples without considering discrepancies between the predicted status and the IHC-based status. As shown in Table 1, in 5%–12% of cases, the predicted status differed from the IHC-based status.

Additionally, for the survival analyses, patients were selected according to the following criteria: (1) pathological cancer stage I, II, or III and (2) age <80 years at initial diagnosis. Subsequently, patients were stratified according to adjuvant drug treatments. The characteristics of the patients included in the survival analyses are summarized in Table 5. 

## 5. Conclusions

Therapeutic decision making in breast cancer is heavily based on the clinical subtype defined by HR and HER2 expression status. NGS-based approaches could allow more accurate characterization of the various molecular and clinical features of breast cancer. GEP-based receptor-status prediction could provide a better understanding of breast cancer pathology and guide physicians in decision making. To improve the performance of GEP-based prediction models, data from larger cohorts are required for standardization of the procedure. In addition, a more comprehensive analysis of receptor status should be performed to identify the characteristics that affect the positivity or negativity of the status of the three receptors, as well as the mechanisms responsible for the discordance between intrinsic subtype and clinical subtype.

## Figures and Tables

**Table 1:** Summary of mismatch rates and predictor genes for ER, PR, and HER2 status prediction.

Item	Mismatch Rate [%] *		Predictor Genes
	TCGA	METABRIC	
ER	6.28	6.26	*ESR1, AGR3, C1orf64, C4orf7, CLEC3A, SOX11, TFF1*
PR	11.43	5.54	*PGR, AGR3, ESR1, NAT1, PVALB, S100A7*
HER2	11.85	5.17	*ERBB2, CPB1, GSTT1, PROM1*

* Between the immunohistochemistry (IHC)-based and the predicted receptor status. Abbreviations: estrogen receptor (ER), progesterone receptor (PR), human epidermal growth factor receptor 2 (HER2), The Cancer Genome Atlas (TCGA), Molecular Taxonomy of Breast Cancer International Consortium (METABRIC).

**Table 2:** HR and HER2 status for each intrinsic subtype as determined by (a) IHC- and (b) GEP-based prediction. Patients with no available IHC-based receptor status were excluded.

Dataset	Subtype	(a) IHC-Based Characterization		(b) GEP-Based Prediction	
		HR+/−	HER2+/−	HR+/−	HER2+/−
TCGA	Luminal A	222/4	24/130	229/2	4/227
	Luminal B	126/1	22/69	127/0	8/119
	Basal-like	16/78	6/59	10/87	2/95
	HER2-enriched	32/24	40/10	44/14	39/19
METABRIC	Luminal A	680/6	19/283	696/0	19/677
	Luminal B	465/1	23/171	474/0	29/445
	Basal-like	61/243	14/118	40/268	24/284
	HER2-enriched	119/111	50/34	125/111	119/117
	Normal breast-like	161/21	11/51	165/19	11/173

IHC: immunohistochemistry; GEP: gene expression profiling.

**Table 3:** A summary of the hazard ratios and associated statistical significance obtained from survival analyses using IHC-based receptor status (IHC) or the predicted status (pred.). For the survival analysis, data from the TCGA and METABRIC datasets were used.

Patient Group		Conditions Compared	# of Samples		*p*-Value		Hazard Ratio	
			IHC	Pred.	IHC	Pred.	IHC	Pred.
**TCGA**								
(a)	HR+	H vs. NH	727 (438, 289)	735 (430, 305)	0.00031	2.11⋅10^−05^	0.89	1.0
(b)	Hormone therapy	HR+ vs. HR–	449 (438, 11)	449 (430, 19)	3.15⋅10^−08^	3.38⋅10^−07^	2.23	2.0
(c)	HR+ in HER2e/Basal	H vs. NH	44 (23, 21)	50 (21, 29)	0.48	0.045	0.65	1.88
(d)	HER2+	T vs. NT	150 (22, 128)	77 (18, 59)	0.021	0.042	19.4	19.6
**METABRIC**								
(e)	HR+	H vs. NH	564 (477, 87)	566 (470, 96)	0.76	0.12	0.06	0.28
(f)	Hormone therapy	HR+ vs. HR–	511 (477, 34)	511 (470, 41)	0.18	0.047	0.36	0.49
(g)	HR+ in HER2e/Basal	H vs. NH	73 (55, 18)	71 (48, 23)	0.66	0.022	0.18	0.77

HR: hormone receptor; H: with hormone therapy regardless of chemotherapy; NH: without hormone therapy; T: with targeted molecular therapy regardless of hormone/chemotherapy; NT: without targeted molecular therapy.

**Table 4:** A summary of data availability in the TCGA BRCA cohort and METABRIC dataset.

Item	TCGA BRCA Cohort	METABRIC	Comment
Gene expression profile	Yes	Yes	
PAM50-based subtype	Yes (partially)	Yes	
ER status	Yes (IHC)	Yes (IHC, non-IHC)	Used IHC-based status
PR status	Yes (IHC)	Yes (non-IHC)	Used for receptor status
HER2 status	Yes (IHC)	Yes (IHC, non-IHC)	Used IHC-based status
RPPA measurements	Yes	No	
Types of drug treatment	Chemo, hormone and targeted molecular therapy	Chemo and hormone therapy	Used for survival analysis
Age at initial diagnosis	Yes	Yes	Used for sample selection
Pathological stage	Yes	Yes	Used for sample selection

PAM50: prediction analysis of microarray 50 genes; RPPA: reverse phase protein array; ER: estrogen receptor; PR: progesterone receptor; HER2: human epidermal growth factor receptor 2.

**Table 5:** A summary of the samples available in the TCGA and METABRIC datasets.

Variable	Conditions	The Number of Available Samples	
		In TCGA	In METABRIC
Age	≤80 years	1039	1783
Pathologic stage:	I	170	464
	II	598	736
	III	232	105
Therapy applied:	Chemotherapy	578	393
	Hormone therapy	495	1084
	Both chemo- and hormone therapy	324	181
	Targeted molecular therapy	30	NA
ER status:	Positive	760	1339
	Negative	230	418
	NA	2	0
PR status:	Positive	663	946
	Negative	324	837
	NA	4	0
HER2 status:	Positive	159	114
	Negative	524	647
	NA	182	27

ER: estrogen receptor; PR: progesterone receptor; HER2: human epidermal growth factor receptor 2. For ER, PR, and HER2 status; ‘indeterminate’ and ‘equivocal’ were reported as NA.

## Supplementary Materials

The following are available online at https://www.mdpi.com/2072-6694/12/5/1165/s1, The following materials contain some of TCGA and METABRIC clinical data and the new predictions on the 3-receptor status, which were used for the survival analyses in this work: Table S1. TCGA_BRAC_clinical_data_*n*_pred_status.csv, Table S2. METABRIC_clinical_data_*n*_pred_status.csv. Figure S1: UMAP plot showing receptor status of patients in the METABRIC dataset. The tumor subtype and ER, PR, and HER2 status were based on the available clinical data. Gray points are samples with no available clinical information. The UMAP plot of the METABRIC dataset revealed a similar macroscopic landscape to that for TCGA. Figure S2: UMAP plot showing receptor status of patients in the METABRIC dataset. GEP-based prediction was used to determine the subtype, as well as the status of ER, PR, and HER2. Similar to TCGA, the predicted ER and HER2 status (but not PR) was mostly in accordance with the corresponding pattern of receptor status for the basal-like, luminal A, and luminal B subtypes. Figure S3: Scatter plots and Pearson’s correlation coefficients of seven predictor genes for ER status prediction in the TCGA BRCA cohort (a) and METABRIC dataset (b). Blue: ER+; red: ER–; empty circle: NA. ER status characterization was based on IHC. Figure S4: Scatter plots and Pearson’s correlation coefficients of six predictor genes for PR status prediction in the TCGA BRCA cohort (a) and METABRIC dataset (b). Blue: PR+; red: PR–; empty circle: NA. The PR status of TCGA samples was based on IHC, whereas that of METABRIC samples was not based on IHC. The PR-status predictor genes included *ESR1* and *AGR3,* which were also predictor genes for ER status. Figure S5: Scatter plots and Pearson’s correlation coefficients of four predictor genes for HER2 status prediction in the TCGA BRCA cohort (a) and METABRIC dataset (b). Blue: HER2+; red: HER2–; empty circle: NA. *CPB1, GSTT1,* and *PROM*1 showed weak correlations with *ERBB2*, implying that HER2 status prediction was determined predominantly by *ERBB2.*
