# Supplementary material for: Hormone Receptor-Status Prediction in Breast Cancer Using Gene Expression Profiles and Their Macroscopic Landscape
Source: Cancers (Basel). 2020 May 5;12(5):1165. doi: 10.3390/cancers12051165 (PMC7281553; doi:10.3390/cancers12051165)
Supplement: Supplementary file 1 [file cancers-12-01165-s001.pdf]

*Supplementary Materials*

# Hormone Receptor-status Prediction in Breast Cancer Using Gene Expression Profiles and Their Macroscopic Landscape

**Seokhyun Yoon, Hye Sung Won, Keunsoo Kang, Kexin Qiu, Woong June Park and Yoon Ho Ko**

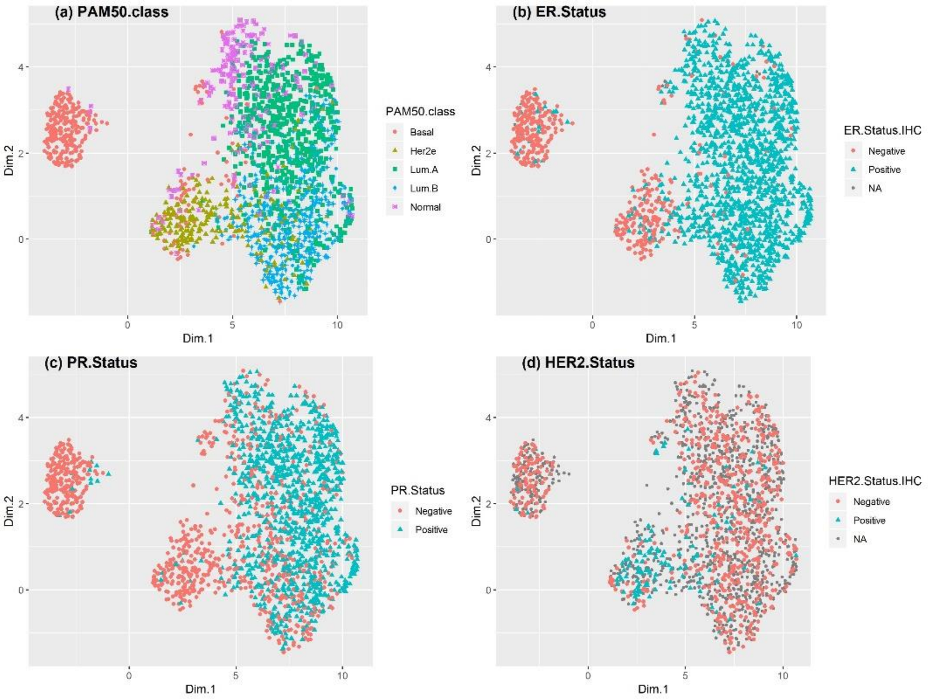


**Figure S1.** UMAP plot showing receptor status of patients in the METABRIC dataset. The tumor subtype and ER, PR, and HER2 status were based on the available clinical data. Gray points are samples with no available clinical information. The UMAP plot of the METABRIC dataset revealed a similar macroscopic landscape to that for TCGA.

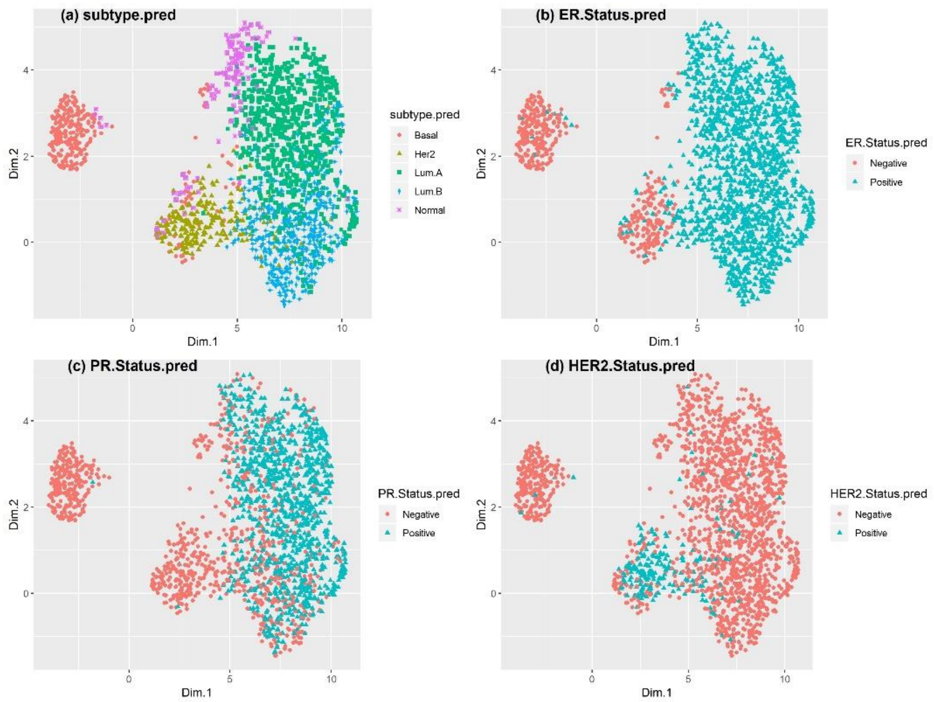


**Figure S2.** UMAP plot showing receptor status of patients in the METABRIC dataset. GEP-based prediction was used to determine the subtype, as well as the status of ER, PR, and HER2. Similar to TCGA, the predicted ER and HER2 status (but not PR) was mostly in accordance with the corresponding pattern of receptor status for the basal-like, luminal A, and luminal B subtypes.

**a.** **b.**

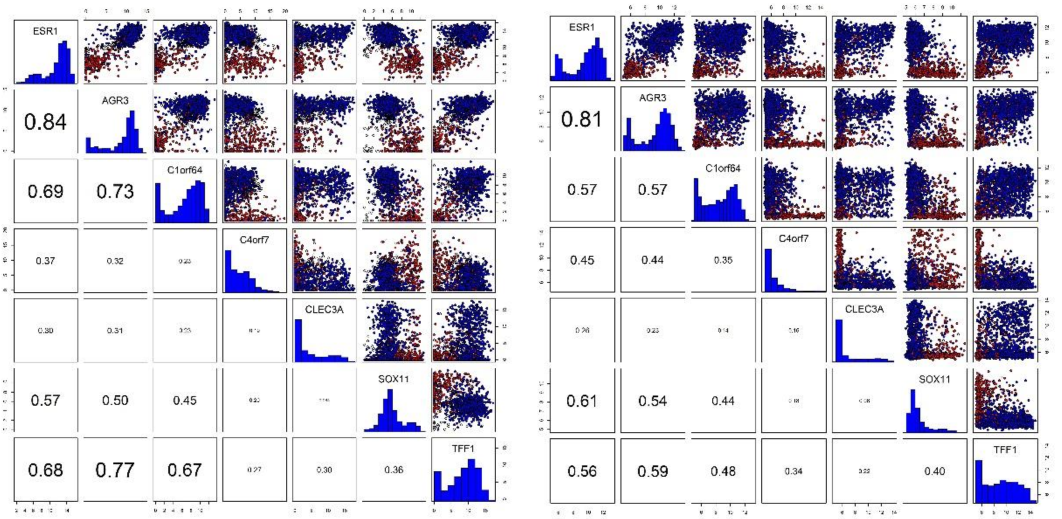


**Figure S3.** Scatter plots and Pearson's correlation coefficients of seven predictor genes for ER status prediction. Blue: ER+; red: ER–; empty circle: NA. ER status characterization was based on IHC.

**a.** **b.**

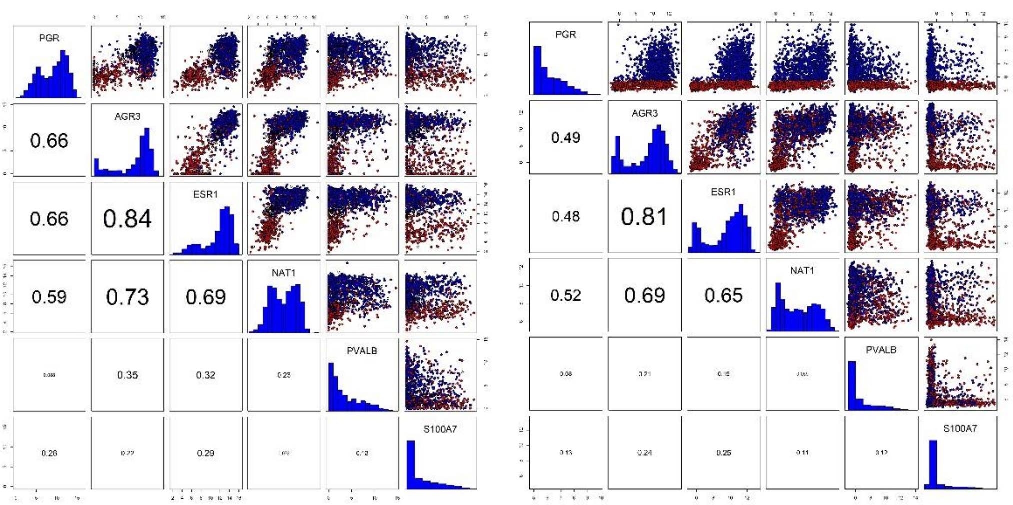


**Figure S4.** Scatter plots and Pearson's correlation coefficients of six predictor genes for PR status prediction. Blue: PR+; red: PR−; empty circle: NA. The PR status of TCGA samples was based on IHC, whereas that of METABRIC samples was not based on IHC. The PR-status predictor genes included ESR1 and AGR3, which were also predictor genes for ER status.

**a.** TCGA **b.** METABRIC

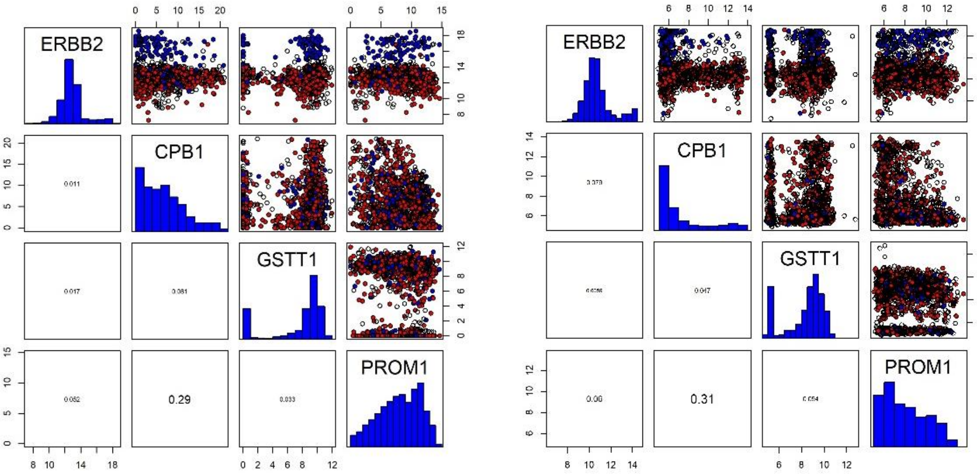


**Figure S5.** Scatter plots and Pearson's correlation coefficients of four predictor genes for HER2 status prediction. Blue: HER2+; red: HER2−; empty circle: NA. CPB1, GSTT1, and PROM1 showed weak correlations with ERBB2, implying that HER2 status prediction was determined predominantly by ERBB2.

© 2019 by the authors. Licensee MDPI, Basel, Switzerland. This article is an open access article distributed under the terms and conditions of the Creative Commons Attribution (CC BY) license (http://creativecommons.org/licenses/by/4.0/).
